# Supplementary figures and images for: Sample-to-answer, extraction-free, real-time RT-LAMP test for SARS-CoV-2 in nasopharyngeal, nasal, and saliva samples: Implications and use for surveillance testing
Source: PLoS One. 2022 Feb 25;17(2):e0264130. doi: 10.1371/journal.pone.0264130 (PMC8880874; doi:10.1371/journal.pone.0264130)

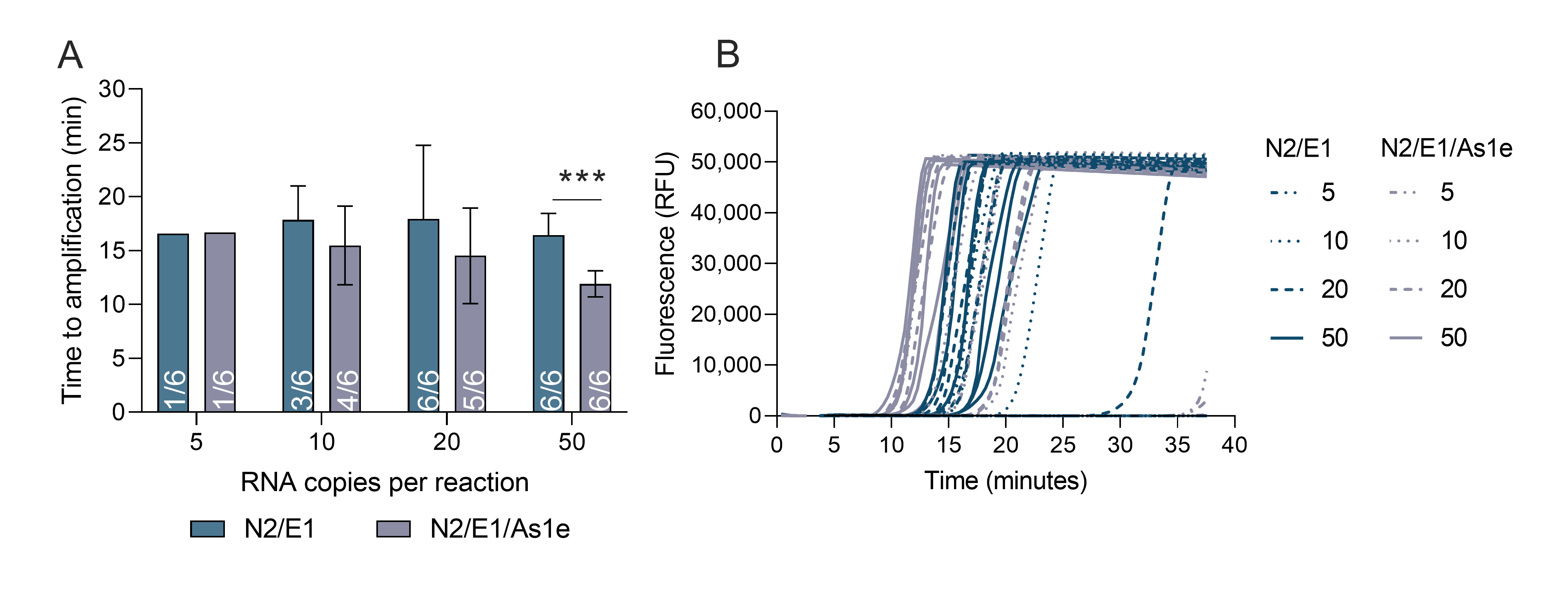

Supplement: S1 Fig — Synthetic SARS-CoV-2 RNA was prepared in nuclease-free water and amplified in RT-LAMP on the Bio-Rad CFX96 using multiplexed primer sets N2/E1 or N2/E1/As1e. (A) Average time to amplification by number of input copies and primer set. The number of replicates that amplified out of 6 is indicated on each bar. Error bars indicate standard deviation of replicates that amplified. (B) Real-time amplification curves for 5, 10, 20, and 50 input copies of RNA per reaction. ***indicates p≤.001; significance determined using an unpaired two-tailed t-test. (TIF) [file pone.0264130.s001.tif]

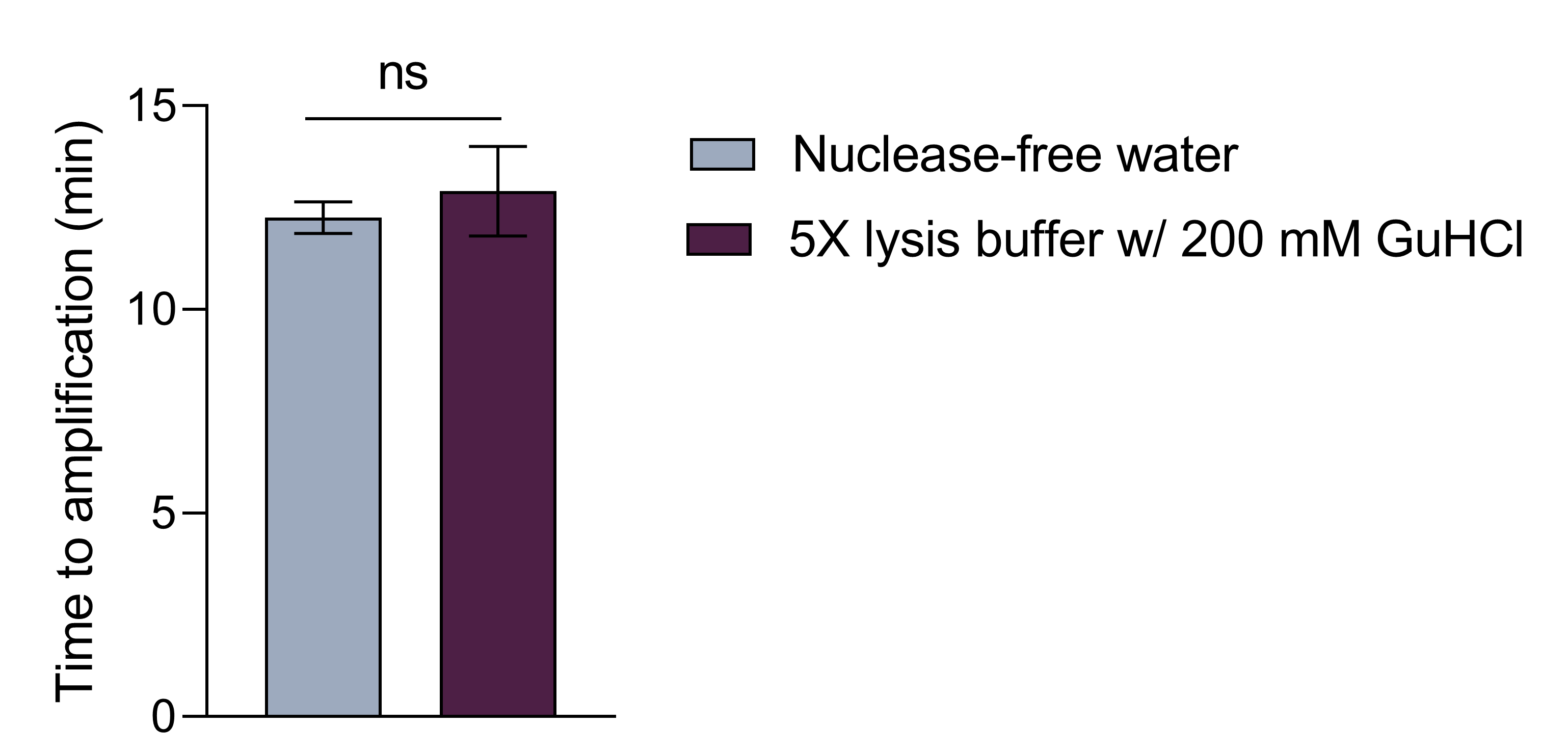

Supplement: S2 Fig — Synthetic SARS-CoV-2 RNA was prepared in either nuclease-free water or 5X lysis buffer with 200 mM GuHCl at a concentration of 10 copies/μL (50 copies per reaction) and amplified in RT-LAMP on the Bio-Rad CFX96. Average time to amplification ± standard deviation is shown (n = 3 for each condition). Time to amplification is not significantly different between the two conditions (p≥0.6; significance determined using unpaired two-tailed t-test). (TIF) [file pone.0264130.s002.tif]

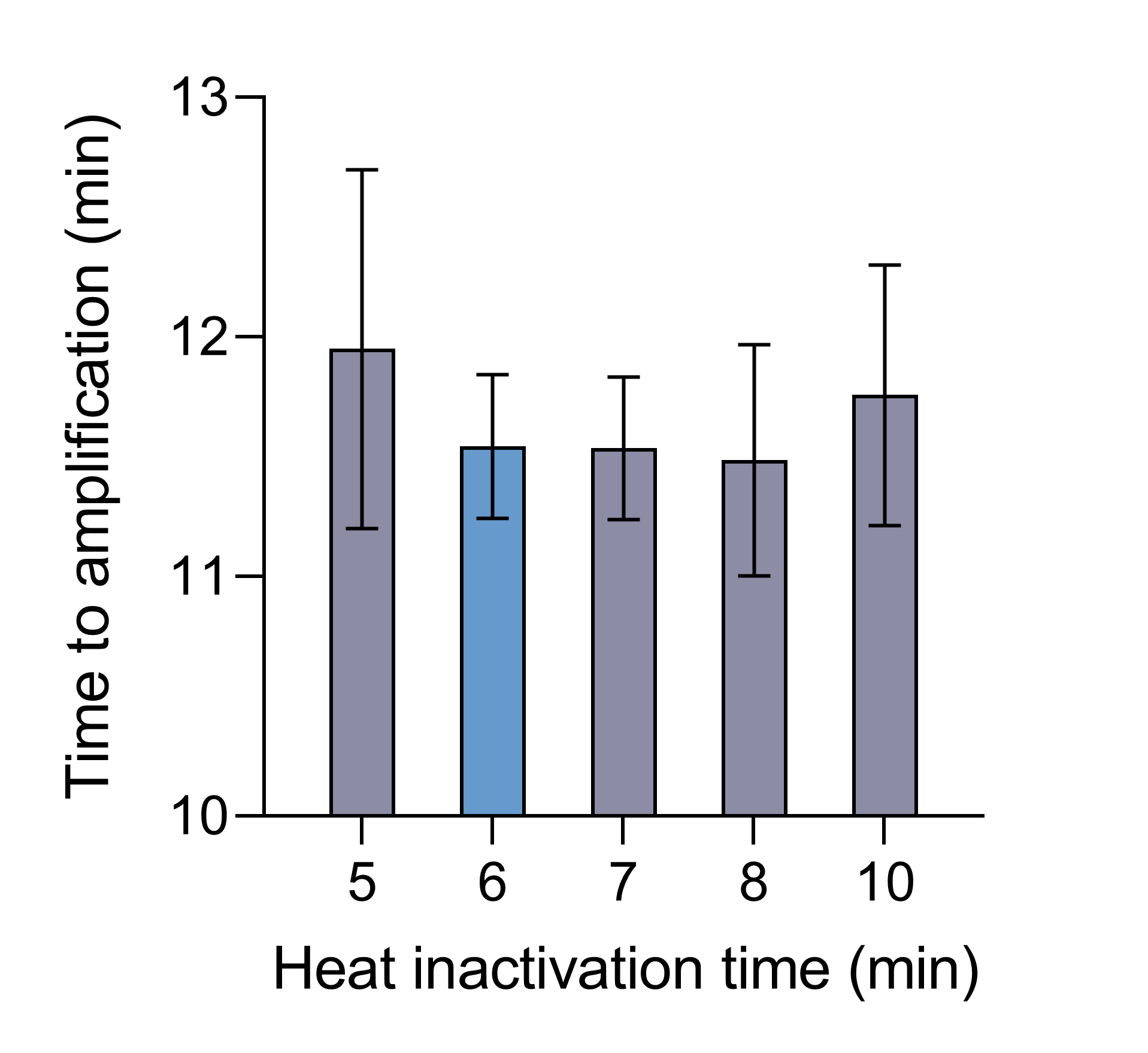

Supplement: S3 Fig — Negative pooled saliva was spiked with Zeptometrix pseudovirus to achieve a concentration of 100 copies/μL, then combined with lysis buffer and heat inactivated at 95°C for 5–10 minutes. The resulting lysate was amplified in RT-LAMP on the Bio-Rad CFX96. Average time to amplification was not statistically different between the heat inactivation time conditions (n = 6 per condition; p = 0.72 determined by one-way ANOVA) but variability of time to amplification was reduced at 6 and 7 minutes of heating. (TIF) [file pone.0264130.s003.tif]

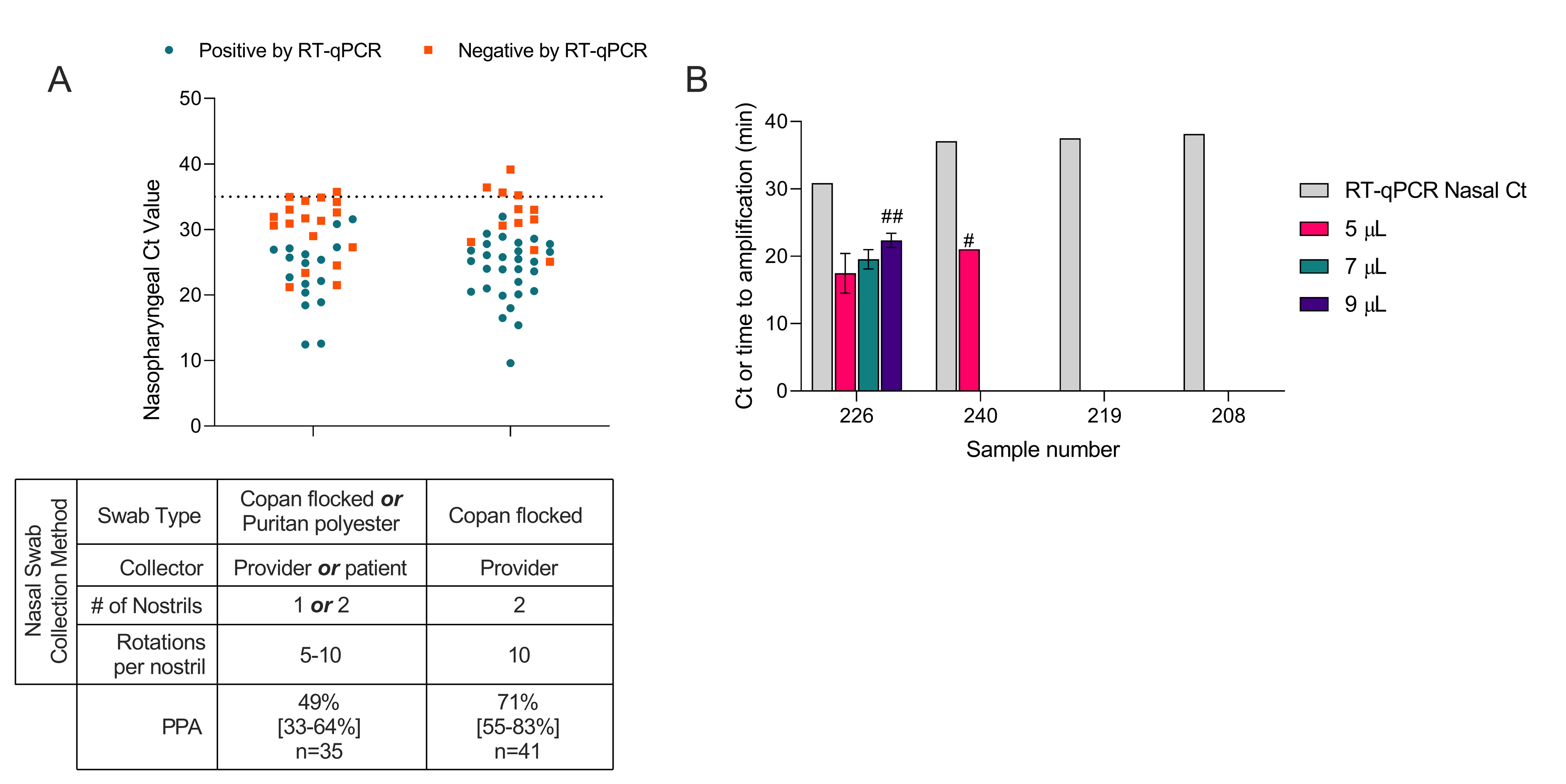

Supplement: S4 Fig — (A) Collection method optimization. Comparison of RT-qPCR results on nasal samples collected with various swabs and swabbing strategies. Nasal RT-qPCR results are stratified by the Ct value of the paired nasopharyngeal swab. (B) Ct (RT-qPCR) or average time to amplification in RT-LAMP (5 μL, 7 μL, 9 μL, indicating sample volume; n = 3 for each condition) of a subset of nasal samples that were initially discordant with RT-qPCR or inconclusive in RT-LAMP with 5 μL samples. Data shown by sample number in order of increasing Ct values; # indicates conditions where only 1 of 3 replicates amplified, ## indicates conditions where 2 of 3 replicates amplified. (TIF) [file pone.0264130.s004.tif]

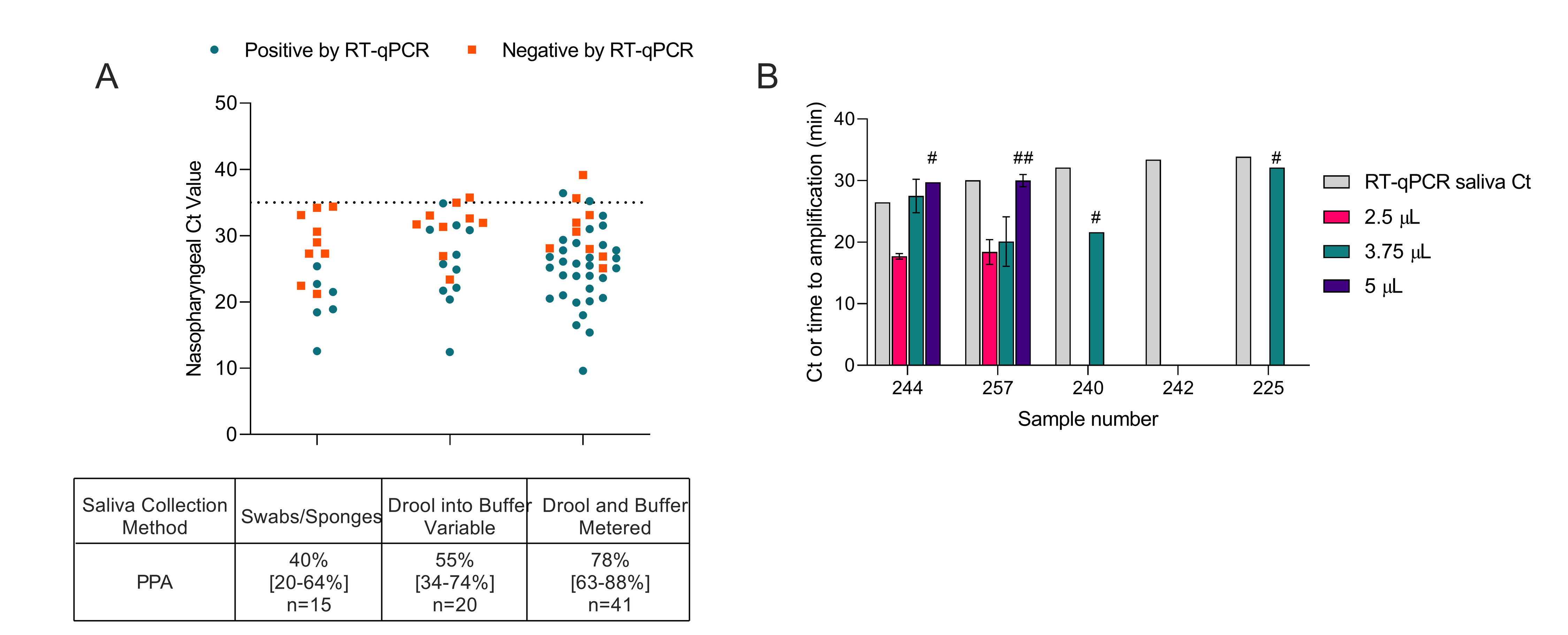

Supplement: S5 Fig — (A) Collection method optimization. Comparison of RT-qPCR results on saliva samples collected with various methods, including by swab or sponge, passive drool directly into buffer resulting in variable saliva to buffer ratios, or metering buffer into drool at a controlled ratio. Saliva RT-qPCR results are stratified by the Ct value of the paired nasopharyngeal swab. (B) Ct (RT-qPCR) or average time to amplification in RT-LAMP (2.5 μL, 3.75 μL, 5 μL, indicating sample volume; n = 3 for each condition) of a subset of saliva samples that were initially discordant with RT-qPCR with 5 μL samples. Data shown by sample number in order of increasing Ct values; # indicates conditions where only 1 of 3 replicates amplified, ## indicates conditions where 2 of 3 replicates amplified. (TIF) [file pone.0264130.s005.tif]

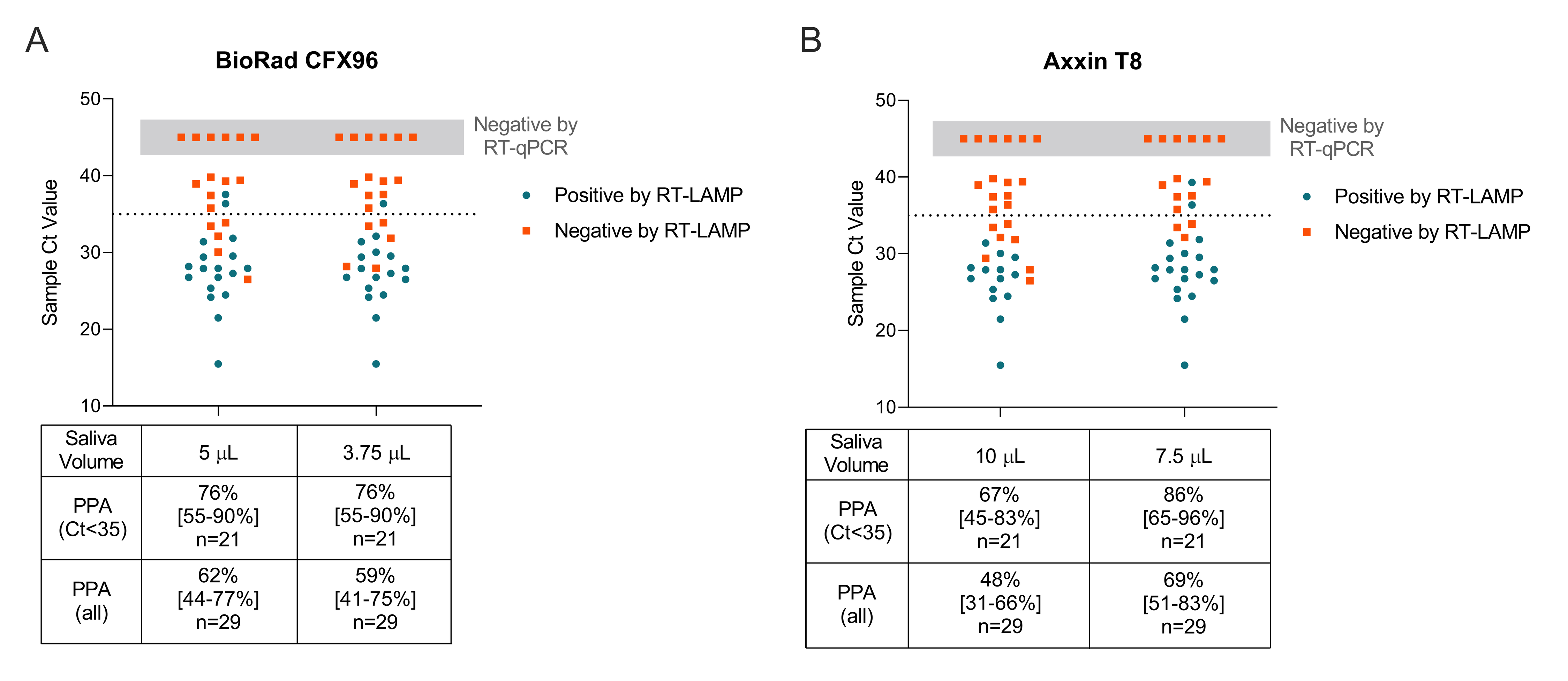

Supplement: S6 Fig — (A) RT-LAMP results for saliva samples tested on the Bio-Rad CFX96, plotted by Ct value of the corresponding RT-qPCR result, using 5 μL sample volume (left) and 3.75 μL sample volume (right), with a table below summarizing positive percent agreement and 95% confidence intervals at different Ct thresholds. (B) RT-LAMP result of saliva samples on the Axxin T8, plotted by Ct value of the corresponding RT-qPCR result, using 10 μL sample volume (left) and 7.5 μL sample volume (right), with a table below summarizing positive percent agreement and 95% confidence intervals at different Ct thresholds. (TIF) [file pone.0264130.s006.tif]

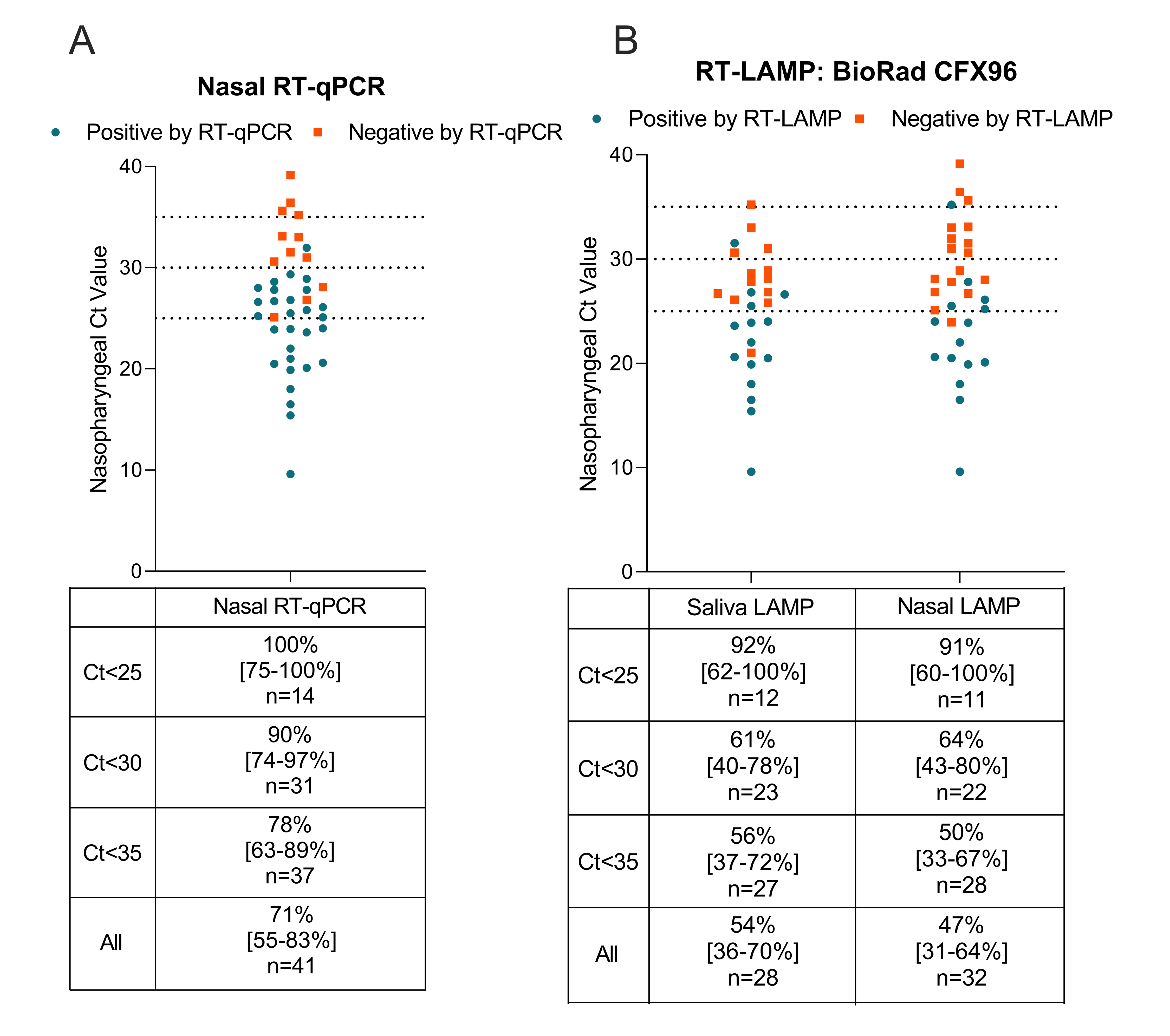

Supplement: S7 Fig — (A) Nasal RT-qPCR results are stratified by the Ct value of the paired nasopharyngeal swab. The table below the graph summarizes percent agreement and 95% confidence intervals at different Ct thresholds. (B) Saliva LAMP and Nasal LAMP results from samples tested on the Bio-Rad CFX96 are stratified by the Ct value of the paired nasopharyngeal swab, with tables summarizing percent agreement and 95% confidence intervals at different Ct thresholds. (TIF) [file pone.0264130.s007.tif]

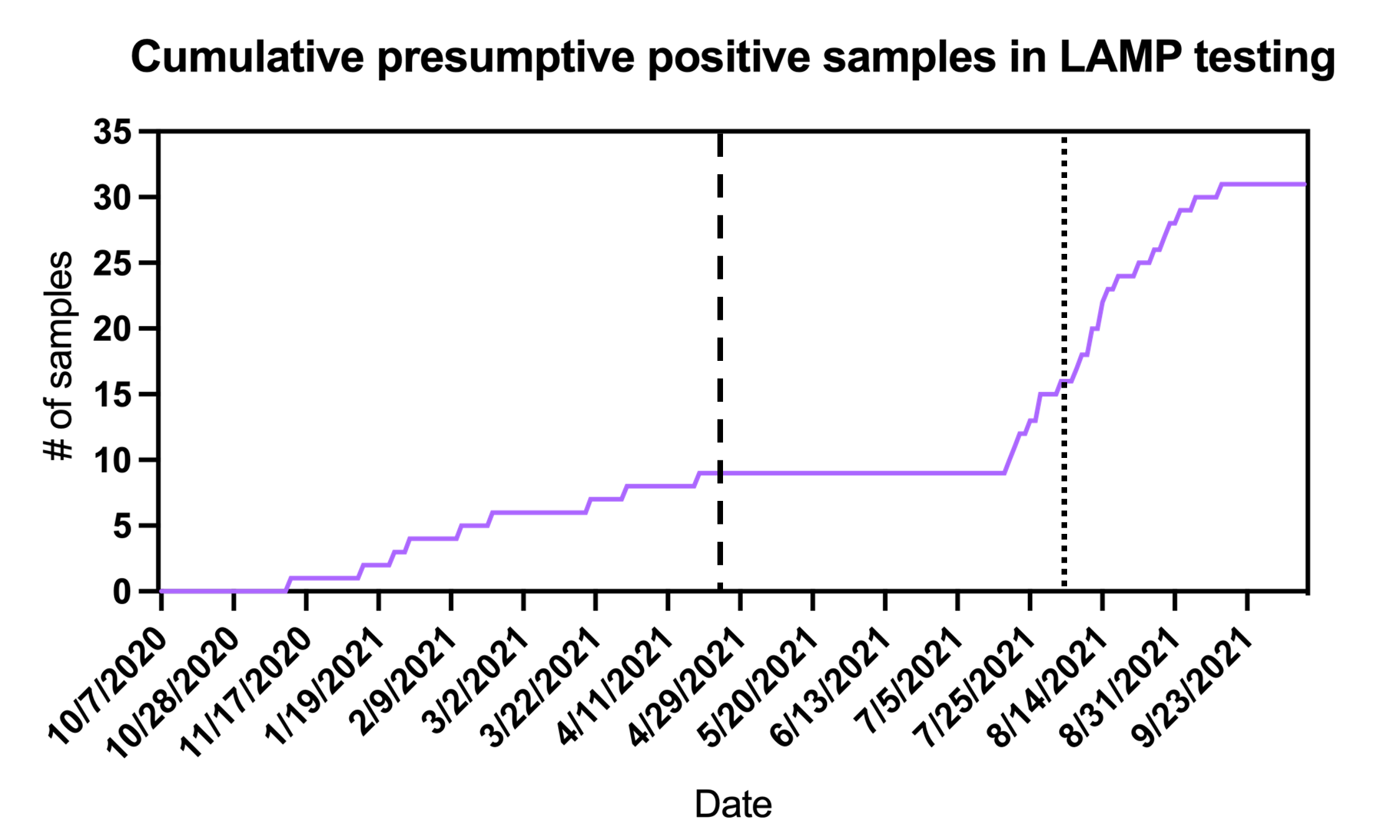

Supplement: S8 Fig — Cumulative number of samples with presumptive positive results by date. Dashed line: university guidance that fully vaccinated individuals no longer need to test (23 April 2021); dotted line: university guidance that fully vaccinated individuals should resume testing (3 August 2021). (TIF) [file pone.0264130.s008.tif]

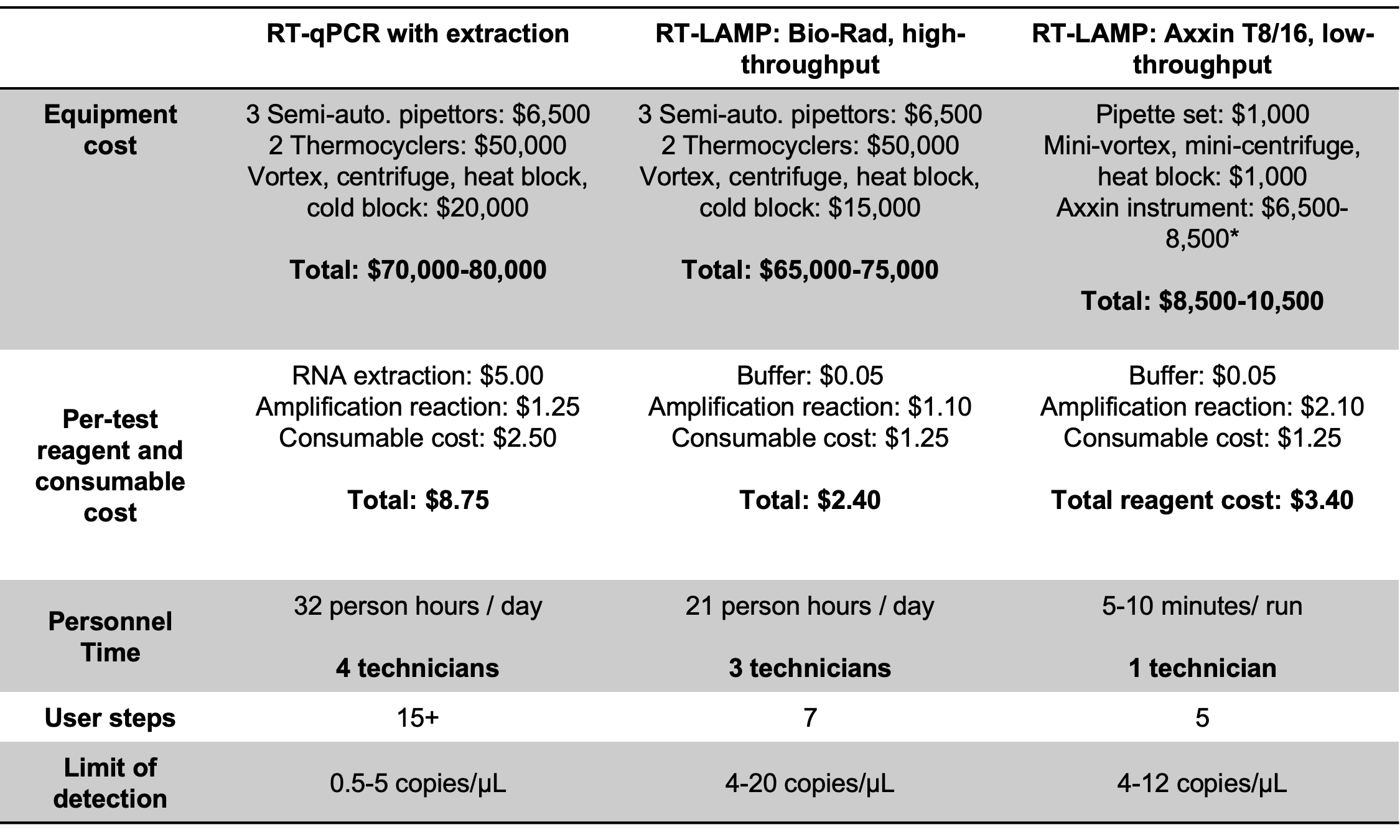

Supplement: S1 Table — Summary of estimated equipment costs, reagent and consumable costs, personnel time, user steps, and limit of detection for RT-qPCR tests and RT-LAMP tests for SARS-CoV-2. (TIF) [file pone.0264130.s009.tif]
