# Supplementary material for: Sample-to-answer, extraction-free, real-time RT-LAMP test for SARS-CoV-2 in nasopharyngeal, nasal, and saliva samples: Implications and use for surveillance testing
Source: PLoS One. 2022 Feb 25;17(2):e0264130. doi: 10.1371/journal.pone.0264130 (PMC8880874; doi:10.1371/journal.pone.0264130)
Supplement: Attachment — Submitted filename: Response to Reviews_v3_clean.pdf [file pone.0264130.s011.pdf]

Dear Dr. Abd El-Aty,

Thank you for the opportunity to revise our manuscript, titled "Sample-to-answer, extraction-free, real-time RT-LAMP test for SARS-CoV-2 in nasopharyngeal, nasal, and saliva samples: Implications and use for surveillance testing." We appreciate the insightful guidance given by the reviewers. Below we explain how we revised our paper after carefully considering their concerns and recommendations.

In short, reviewers requested clarifications on some of the methods. We added the requested details to improve the clarity of our work.

Below, we offer detailed responses to each of the reviewer's comments (our responses in blue). We hope these changes will make the manuscript a valuable contribution to *PLOS One*.

Comments from Reviewer #1:

Overall the manuscript is well written and clear. There are a few sections where some of the methods for comparison are a bit low on numbers or used non-optimal specimens or controls. This does not add any unjustified conclusions, but could have more optimal conditions set. Here are following comments and recommendations for the manuscript.

Major Comments

1. What is the extraction method for the taqpath assay that was used for the RT-PCR reference method? This assay can have a variety of extraction methods that can have large differences on sensitivity.

   We have added the extraction method used to the manuscript.

   Revised Text:

   Pooled negative nasopharyngeal matrix was generated from 10 nasopharyngeal swabs that were confirmed negative by a clinical laboratory using the Cepheid Xpert System SARS-CoV-2 real-time PCR tests, or the Applied Biosystems TaqPath COVID-19 real-time PCR tests **using the MagNA Pure 24 Total NA Isolation Kit (Roche Molecular Systems, 07658036001**).

2. What was the rationale for placing NP into 300uls and Nasal into 500uls. Although small, this dilution factor could cause some of the differences between sensitivity when comparing results.

We aimed to place swabs into as little volume as possible in order to dilute the virus as little as possible; however, due to the shape of the swabs, a different tube and slightly more volume was required to fully submerge the nasal swabs compared to the NP swabs. We anticipate minor differences in sensitivity but anticipate those would be on the same order of magnitude and would not change any conclusions. We have added a note in the text regarding this.

Revised Text:

Methods: Nasopharyngeal swabs were collected into 1.5 mL sterile Sarstedt screw-top tubes (Nümbrecht, Germany) containing 300 µL of 5X lysis buffer. Nasal samples were collected into 2 mL sterile Sarstedt screw-top tubes containing 500 µL of 5X lysis buffer. **Due to the shape of the swabs, a different tube and slightly more volume was required to fully submerge the nasal swabs compared to the nasopharyngeal swab.**

Results**: Although nasal swabs required a slightly higher volume to submerge than nasopharyngeal swabs (500 µL vs. 300 µL), we anticipate that any differences in sensitivity attributed to this would be on the same order of magnitude and likely negligible.**

3. The addition of multiple freeze thaws can cause degradation of specimens. Generally, a specimens should not go through more than 1 freeze thaw. Was any testing performed to determine that the CT values did not increase after each freeze thaw.

   We appreciate this concern from the reviewer. We did perform testing to determine the effect of three freeze/thaw cycles on both purified and unpurified samples and we determined that viral RNA remained stable through three freeze/thaw cycles. There is good evidence published now, summarized below and now cited in the text, that indicates viral RNA levels are relatively stable at repetitive freeze-thawing.

   - Repetitive freeze-thaw cycles increase Ct values, although only moderately and not to the degree that it will substantially alter the viral detectability [J Mol Diagn 2021, 23: 691-697; https://doi.org/10.1016/ j.jmoldx.2021.03.003]
   - Changes in Ct values were analyzed after storage in different conditions and it was found that Ct values increased after storage or treatment in all the above conditions. Taking the amplification of ORF1ab as an example, the influence of freeze-thaw cycles was relatively small, with an increase in Ct of 0.41 for 1 freeze-thaw cycle and 0.82 for 2 cycles. [Journal of Infections Diseases 2020, 222; 10.1093/infdis/jiaa272]

Revised Text:

The unused portion of all samples was stored at -80 ºC, and samples used for retesting underwent a maximum of 3 freeze-thaw cycles **[J Mol Diagn 2021, 23: 691-697; https://doi.org/10.1016/ j.jmoldx.2021.03.003]**.

4. What was the rationale for testing surveillance specimens in triplicate? Was this to increase sensitivity due to issues found in previous experiments?

   To clarify, only suspected positives in the surveillance population were tested in triplicate, and an algorithm that included melt curve derivative analysis was applied, detailed in "Bio-Rad CFX96 RT-LAMP data" and pasted below. The rationale for this was not to increase sensitivity but to improve specificity, because LAMP is prone to nonspecific amplification. When samples with nonspecific amplification are re-measured in triplicate, we expect a very low chance of samples amplifying again; however, when true positives are re-measured in triplicate, we expect to see consistent amplification. By repeating testing in triplicate as well as analyzing melt curve derivatives, we increased confidence in true positive identification.

   Algorithm described in text ("Bio-Rad CFX96 RT-LAMP Data"):
   Samples tested in triplicate were considered positive if all three replicates were given a Ct value, or negative if none of the three replicates were given a Ct value. If one or two of three replicates were given a Ct value, the following algorithm was applied: (1) If any of the three replicates amplified before 30 minutes (< 81 cycles), the sample was considered positive. (2) If at least one of the three replicates amplified between 30 and 45 minutes (81-121 cycles), both of the following conditions had to be met for the sample to be considered positive: (A) the melt curve derivative peak of at least one replicate was within 1 ºC of the melt curve derivative peak of a positive control run on the same plate and (B) the melt curve derivative remained positive between 65-70 ºC. (3) Otherwise, the sample was considered negative.

5. Testing a subset of specimens for internal controls is not a traditional method. What was the number of specimens that failed and how often was re-testing needed.

   Because this is a surveillance program, we tested a subset of samples in the beta actin assay as a process control and plate level control rather than a per-sample control. The metrics tracked over time included the average time to amplification of all samples tested in the beta actin assay (purple squares), and the percent of samples that did not amplify on a given day of testing (blue dots).

   Revised Text:

With every run of the SARS-CoV-2 assay, a subset of participant samples was also tested in the beta actin RT-LAMP assay as a **plate and process** control **designed to help identify issues like sample degradation during transport, insufficient lysis, or LAMP reagent quality deterioration**. If greater than 20% of participant samples included in the beta actin assay failed to amplify, lysis buffer was discarded and re-prepared with fresh reagents.

6. For the surveillance testing, was all specimens also tested on a RT-PCR as a reference method. If so this comparison should be highlighted to discuss PPA and NPA.

No, all specimens were not tested on RT-qPCR because this was a surveillance testing program. However, surveillance testing was only implemented on a large scale after thorough evaluation of the RT-LAMP assay with clinical samples, comparing performance of RT-LAMP to a clinical reference RT-qPCR (described in the first part of the paper).

Minor Comments

7. Ln89-103 This section is written as an abstract with data instead of a lead in for the introduction.

Thank you for this feedback. We have revised these lines as follows to remove data and phrase it as a lead in for the introduction.

Revised Text:

Here, we present an RT-LAMP test for SARS-CoV-2 with a sample-to-answer workflow optimized for use with saliva, nasal, or nasopharyngeal swabs that can be performed with a standard thermocycler or portable isothermal fluorimeter. The test uses a simple lysis and inactivation protocol adapted from Rabe and Cepko (17) and provides results in <1 hour using inexpensive and readily available reagents. Test performance was compared to that of RT-qPCR using nasopharyngeal swabs, nasal swabs and saliva obtained from patients hospitalized due to COVID-19. ~~For samples with Ct <30, the optimized RT-LAMP test run on a portable isothermal fluorimeter showed 100% agreement for nasopharyngeal, nasal, and saliva samples.~~ Following test validation, we piloted a saliva-based RT-LAMP testing program at Rice University and transitioned to a surveillance testing program. Between August 2020 and June 2021, up to 400 individuals were tested per day. ~~Out of the 10,789 individuals tested, nine had a positive surveillance test result and were referred for confirmatory testing; eight of those individuals were confirmed positive by nasal swab RT-qPCR.~~ The simple workflow, reliance on more readily available reagents, and effective detection of high viral load samples indicate that the optimized RT-LAMP assay presented here is an effective strategy for SARS-CoV-2 surveillance.

8. Overall the manuscript is a bit long and could have portions removed to make it a more concise manuscript. I would suggest modifying to reduce the length. Some examples that could be removed are parts of the materials and methods for example the NP collection method, methods for reducing cross contamination.

   We believe these sections will be of interest to readers. It is important to convey the difficulty of collecting quality NP swabs. Moreover, because of the highly sensitive nature of LAMP, it is critical to implement techniques to prevent cross contamination.

9. LoD study: The testing that was performed was with a construct quality control material instead of an inactivated virus. Was any testing performed with virus for sensitivity as the construct vs real virus could have downstream effects of extraction methods.

   We were unable to culture virus due to biosafety level restrictions at our university. We considered using true positive samples as positive controls and attempted to titrate true positives for use as controls in early experiments. However, this was not a viable long-term solution due to the rarity of true positives in our population at the beginning of the pandemic. The sample volume needed to perform all the required experiments for assay validation far exceeded that which was available from the true positive samples available to our team at that time. However, the pseudovirus used, NATtrol™ SARS-CoV-2 Stock, is formulated with purified, intact viral particles. The virus particles have been chemically modified to render them non-infectious and refrigerator stable. We believe downstream effects of extraction would not be seen in our assay due to the extraction-free lysis method used. Additionally, the controls used met the requirements for Emergency Use Authorization application by the FDA and have been commonly used in other published SARS-CoV-2 related research.

10. Figure 3 – What was the # of copies per… ml or ul? When looking at the amount of saliva added to the specimens for saliva, what was the starting CT for those specimens, this can be from the NP sample.

    To clarify, in this experiment to determine limit of detection, we spiked pseudovirus into negative matrix. For these specimens the number of copies listed in the figure refers to total number of copies in the reaction. We revised the text to make this clear. We do not have Ct values for the pseudovirus prior to spiking, but we have copy number per µL as reported by the manufacturer, which we used to dilute and add that to the reactions.

    Relevant Text:

    All **reactions contained 5 µL of sample unless otherwise noted**, and reactions were supplemented with nuclease-free water to reach a final reaction volume of 25 µL… For

RT-LAMP reactions run on the Axxin T8-ISO (T8), assay components described above were doubled to achieve 50 µL reaction volumes with two exceptions (36): fluorescent dye was used at a 5X concentration instead of 50X (37), and 0.2 µL of Tte UvrD helicase (NEB) was included with each reaction (38,39)… **Reactions contained 10 µL of sample unless otherwise noted.**

Comments from Reviewer #2:

A well written manuscript with very detail description of the methods and procedures.

11. The use of multiple primer sets can increase sensitivity, but it increases primer cost. Need some information on the cost difference.

    We appreciate the reviewer's concern about increased cost. Since all primers used in this assay are unlabeled and only two out of the six in each set require HPLC purification, the addition of more primer sets results in an almost negligible impact on total assay cost, especially when oligos are purchased at a large scale. When primers are produced at a 10 micromole scale, each primer set costs about $0.07. The total reaction cost with one, two, or three primer sets is $2.26, $2.33, and $2.40, so the change from one and three primer sets only adds (2.40/2.26) ~ 6% to the total cost.

    Revised Text:

    However, the combination of N2, E1, and As1e primer sets in RT-LAMP resulted in faster times to amplification, with a significant difference ($p<0.001$) seen at 50 input copies (S1 Fig). Thus, the triplex RT-LAMP assay was chosen due to its sensitivity, rapid time to amplification, **and negligible increase in cost compared to the duplex RT-LAMP assay**.

12. The authors may want to address cross-contamination issue when the sample preparation step is done manually and pipetted into tubes.

    We appreciate the reviewer's concern about cross-contamination with manual sample preparation. To prevent this we implemented stringent training for laboratory personnel, separate filtered pipette tips for all materials, and frequent changing of gloves. We clarified this in the text. Additionally, we draw attention to the "clean reaction setup" description.

    Revised Text ("Clean reaction setup"):

    All amplification reactions were assembled and sealed prior to amplification in a dedicated pre-amplification room that was regularly decontaminated with bleach and RNaseAwayTM (ThermoFisher) and had limited personnel access. **Stringent training**

was implemented for laboratory personnel, separate filtered pipette tips were used for all materials, and gloves were changed frequently. Once reactions were run, reaction tubes were disposed of, without being opened, to prevent post-amplification contamination of future reactions.

13. The manuscript should address the total cost (including labor) needed to perform each LAMP test in the near POC (using Axxin device) and high throughput (using the Bio-Rad thermal cycle) settings versus real-time RT-PCR tests performed primarily in high throughput lab.

    We have added a table comparing the estimated equipment, reagent, consumable costs to the Supplementary Material (S1 Table, reproduced below). The total reagent/equipment cost (excluding labor) needed to perform each LAMP test in the near POC is $3.40 and high throughput is $2.40. Real-time RT-qPCR with extraction costs about $8.75 per reaction. We find it difficult to factor in labor because it is highly context-specific and is dependent on laboratory infrastructure in the local area, demand for technicians, number of tests performed, and whether that number is below the maximum capacity able to be completed by the staff. For example, during the course of our study we had three different testing policies implemented at our university, which vastly impacted testing demand and associated labor costs.

	**RT-qPCR with extraction**	**RT-LAMP: Bio-Rad, high-throughput**	**RT-LAMP: Axxin T8/16, low-throughput**
**Equipment cost**	3 Semi-auto. pipettors: $6,500<br>2 Thermocyclers: $50,000<br>Vortex, centrifuge, heat block, cold block: $20,000<br><br>**Total: $70,000-80,000**	3 Semi-auto. pipettors: $6,500<br>2 Thermocyclers: $50,000<br>Vortex, centrifuge, heat block, cold block: $15,000<br><br>**Total: $65,000-75,000**	Pipette set: $1,000<br>Mini-vortex, mini-centrifuge, heat block: $1,000<br>Axxin instrument: $6,500-8,500*<br><br>**Total: $8,500-10,500**
**Per-test reagent and consumable cost**	RNA extraction: $5.00<br>Amplification reaction: $1.25<br>Consumable cost: $2.50<br><br>**Total: $8.75**	Buffer: $0.05<br>Amplification reaction: $1.10<br>Consumable cost: $1.25<br><br>**Total: $2.40**	Buffer: $0.05<br>Amplification reaction: $2.10<br>Consumable cost: $1.25<br><br>**Total reagent cost: $3.40**
**Personnel Time**	32 person hours / day<br><br>**4 technicians**	21 person hours / day<br><br>**3 technicians**	5-10 minutes/ run<br><br>**1 technician**
**User steps**	15+	7	5
**Limit of detection**	0.5-5 copies/µL	4-20 copies/µL	4-12 copies/µL

S1 Table. Test comparison. Summary of estimated equipment costs, reagent and consumable costs, personnel time, user steps, and limit of detection for RT-qPCR tests and RT-LAMP tests for SARS-CoV-2.

14. This work confirms the previous findings that samples with low Ct values were more commonly found in the asymptomatic surveillance population than the hospitalized population.

    We agree and included a citation to Walsh et al.

15. The manuscript stated that "sample collection, inactivation, and amplification requires <5 user steps, and results can be obtained in <1 hour." It is unclear if this is for a single sample or the same turn-around-time can be obtained if 8 samples are collected and processed for the Axxin incubator/detector.

    We revised the paper to clarify that this is for a single sample, and 8 samples being collected and processed would add a small additional time. Specifically, the parts that have time added are collection of each sample and inactivation (adding lysis buffer to each sample), and everything else is a batch process that does not add time.

    Revised Text:
    Sample collection, inactivation, and amplification requires <5 user steps **per sample**, and results can be obtained in <1 hour. Moreover, the assay can be adapted to accommodate higher throughputs needed for population surveillance by incorporating automation and real-time thermocycling machines, such as the Bio-Rad CFX96 (Fig 1G-M), **while still maintaining rapid time to result.**

Overall, this manuscript provides readers with information on the success of using LAMP assay to perform surveillance testing in a university campus setting. I'd recommend it for publication with minor revision.

Comments from Reviewer #3:

Thank you for inviting me to peer review the manuscript titled "Sample-to-answer, extraction-free, real-time RT-LAMP test for SARS-CoV-2 in nasopharyngeal, nasal, and saliva samples: Implications and use for surveillance testing" by Kathryn A. Kundrod et al. The authors optimized a RT-LAMP based nucleic acid test for SARS-CoV-2 detection for nasopharyngeal swabs, nasal swabs, and saliva collected from hospitalized patients. They used Hamilton Microlab Prep device to automate the procedure

1. (It should be noted that currently a FDA EUA approved LAMP-based test is also available for home testing, Lucira COVID-19 All-In-One Test Kit, prescribed to be used in PoC and also at a home setting for suspected people older than 14. This is not mentioned in the manuscript).

   We added this context to the manuscript.

   Revised Text:

   Successful implementation and validation of the RT-LAMP assay on the Axxin T8 has overcome the challenges of adapting assays to point-of-care instruments (36) and shows the potential of the use of this workflow near the point-of-care. **FDA Emergency Use Authorization and widespread use of the Lucira COVID-19 All-In-One Test Kit at the time of publication illustrates the potential for similar technologies to be translated for true point-of-care use.**

The manuscript is well written and could be interesting to the readers. After careful reading, I decide to accept this paper for publication.

Comments from Reviewer # 4:

In this study, Kundrod et al. undertake broad validation of RT-LAMP for community-based screening that addresses many fundamental concerns about faster, simpler techniques being developed around the world in the wake of COVID-19. Through laboratory-based comparisons with the clinical standard, detailed description of LOD in different sample types and under various conditions in a clinical cohort, and application in a community-based setting with low incidence, the authors clearly demonstrate the utility of RT-LAMP to detect those with high levels of viral RNA more quickly than the current standard. The study is very well-written, with clearly explained objectives that seem highly relevant to those (like myself) who are new to the field. As a result, I can find very little to criticize about it, and can't wait to apply the lessons described by the authors in my own work.

1. If possible, it would be nice to see some additional discussion about the cost vs. benefit of the surveillance activities undertaken - how much did each of those 9 cases cost? How low does the cost have to be to justify detection of such a small proportion of positives? Practically speaking, won't people just stick with symptom screening and hope for the best, because it is exponentially cheaper?

   We appreciate these important considerations raised by the reviewer. This type of modeling is beyond the scope of our work, but a number of other papers have considered the time interval and acceptable sensitivity of a test used for safe reopening of college campuses

[JASON, "Managing the Risk From COVID-19 During a Return to On-Site University Research https://fas.org/irp/agency/dod/jason/covid-19.pdf; Walensky *et al.*, JAMA Network Open, doi:10.1001/jamanetworkopen.2020.16818]. These studies were the motivation for the campuswide surveillance of which LAMP was a part. Also, since the initial submission of our manuscript, a notable increase in presumptive positives, shown below, indicated the delta wave on campus/in Houston, and helped inform changes to campus policies including a reinstation of the mask mandate. We added test data through October 11, 2021, which brings our total to 20,645 total tests and 31 positives, and added the below graph to the manuscript (S8 Fig). We also note that our testing was not the source of the August 2021 false positives that were covered in the New York Times and other news outlets.

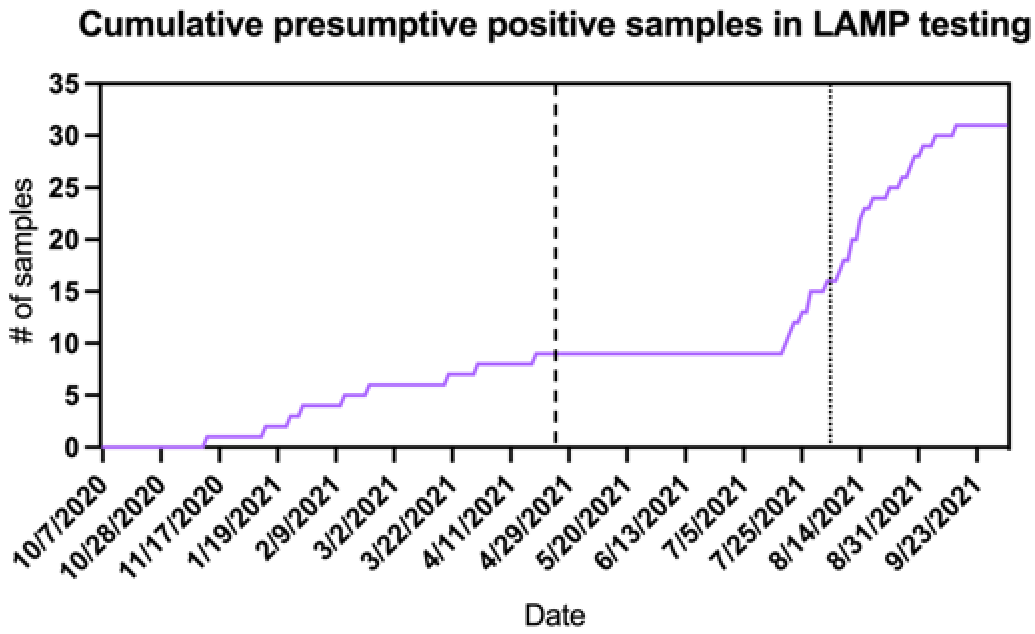

S8 Fig. Cumulative presumptive positive samples in LAMP testing. Cumulative number of samples with presumptive positive results by date. Dashed line: university guidance that fully vaccinated individuals no longer need to test (23 April 2021); dotted line: university guidance that fully vaccinated individuals should resume testing (3 August 2021).

2. The point about repeat testing vs. sensitivity is well-taken, since someone could have a low viral load just prior to peak infectivity. But information about how many asymptomatic individuals were tested how many times is not given. Individuals were tested weekly or bi-weekly, but based on your experience what would be the ideal time interval between samples, to minimize the amount of samples required to monitor a given population? Just out of curiosity, not because I think the paper requires any major revision.

We appreciate this interesting question raised by the reviewer. An analytical modeling study performed by Walensky *et al.*, cited in the above response, concluded that symptom-based screening alone was not sufficient to contain an outbreak, and screening every two days was the best way to control outbreaks at a justifiable cost. Unfortunately, we don't have the full picture regarding total individuals tested at Rice University, and their compliance to testing requirements, as we were only one of three sites providing surveillance testing. Rice chose to adapt testing frequency dynamically throughout the pandemic; the policy varied based on changes throughout the pandemic, with one of the main metrics of success being low on-campus transmission. For example, the policy began as weekly testing for all individuals on campus; when high transmission was seen in Houston, the policy changed to twice weekly; post-vaccine, vaccinated individuals did not have to test until the Delta wave reached Houston, at which point vaccinated individuals had to test weekly and unvaccinated twice weekly. The right time interval may require dynamic adjustment based on: rates of transmission, vaccination status, mask mandates, and speed/effectiveness of contact tracing. Rates of on-campus transmission remained near zero until the Delta wave and still remained low after that.
