# Supplementary material for: Sample-to-answer, extraction-free, real-time RT-LAMP test for SARS-CoV-2 in nasopharyngeal, nasal, and saliva samples: Implications and use for surveillance testing
Source: PLoS One. 2022 Feb 25;17(2):e0264130. doi: 10.1371/journal.pone.0264130 (PMC8880874; doi:10.1371/journal.pone.0264130)
Supplement: Attachment — Submitted filename: Response to Reviews 2_v3_clean.pdf [file pone.0264130.s012.pdf]

Dear Dr. Abd El-Aty,

Thank you for the opportunity to further revise our manuscript, titled "Sample-to-answer, extraction-free, real-time RT-LAMP test for SARS-CoV-2 in nasopharyngeal, nasal, and saliva samples: Implications and use for surveillance testing." We have revised our paper to address our reviewer's remaining concerns and recommendations.

Below, we offer detailed responses to each of the reviewer's comments (our responses in blue). We hope these changes will make the manuscript a valuable contribution to *PLOS One*.

Comments from Reviewer #1:

The manuscript submitted by Richards-Kortum et al entitled "Samples-to-answer, extraction-free real-time RT-LAMP test for SARS-CoV-2 in NP, NS, and saliva samples: Implications for surveillance testing" describes a study where the authors developed a RT-LAMP assay for detection of SARS-CoV-2 from NP, NS, and saliva samples. The assay uses 3 viral targets and an internal human control. Initially they optimized the assay for extraction and amplification using synthetic controls. When optimized they validated the assay with previous positive hospitalized patients. Results from these studies are similar with others where NP was most sensitive followed by NS and saliva samples. Also similar to other publications isothermal LAMP showed a reduced sensitivity compared to traditional RT-PCR assays. The authors then used the assay for a local screening of 400 individuals. A subset of positives was confirmed positive by a RT-PCR instrument. Overall, the manuscript is well written; however, these data are similar to other assays on the market. The most novel aspect is the surveillance testing, which could use some additional details and clarity. Prior to submission the following comments and suggestions should be addressed.

Major Comments

1. Validation patient samples were all identified as previous positive patients which can skew results for the validation due to a higher-than-average prevalence. I would suggest increasing the number of negative samples tested to adequately evaluate the specificity of the assay or at minimum discuss this as a limitation.

   To clarify, in addition to the 69 positive nasopharyngeal, 42 positive nasal, and 35 positive saliva samples we used for validation, we also obtained validation RT-qPCR tests on 5% of the negative samples tested during the pilot phase of scaling up high-throughput RT-LAMP saliva testing. Of the 116 negative samples tested in confirmatory RT-qPCR, 100% were confirmed negative. We have added these results in the revised text that follows.

Revised Text:

As of December 16, 2021, a total of 24,927 participants at Rice University were tested in the high-throughput RT-LAMP assay. Thirty-eight people had presumptive positive test results, and the remainder of the participants tested presumptively negative. ~~Presumptive positive test results increased dramatically upon the arrival of the delta variant to Houston (S8 Fig), and the trend seen in LAMP testing presumptive positivity was used in part to inform new guidance on masks and gathering size.~~ Individuals who tested positive by saliva RT-LAMP**, and five percent of individuals who tested negative by saliva RT-LAMP between September 2020 and January 2021,** were directed to undergo a follow-up confirmatory nasal RT-qPCR within 24-48 hours. Data were available for nine of the 38 confirmatory tests, which were performed by one of Rice University's contracted COVID-19 testing providers on a self-collected nasal sample. Eight received a positive confirmatory test result and one received a negative confirmatory test result. The research team did not have access to laboratory results for the other 22 positive confirmatory tests which were performed by non-Rice affiliated community providers. **All negative validation tests (n=116) were confirmed negative.**

2. Figure 7: In the abstract the follow up screening was performed using 400 individuals, but the numbers in the text ln668 had 20,645 and all of these seem to be nasal and not saliva.

   To clarify, we tested up to 400 individuals per day, not in total. Over the duration of surveillance testing through October 11, 2021, the total number of tests performed (≤400 per day) summed to 20,645. No changes were made to the text on this point. The 20,645 samples collected for routine RT-LAMP testing were saliva, not nasal samples. Please note that we have updated the manuscript to include data through December 16, 2021, so now the total is 24,927 participants. Confirmatory RT-qPCR tests on presumed positive individuals identified from RT-LAMP were run using nasal swabs. Figure 7 describes the results of the confirmatory nasal RT-qPCR tests that were run for all individuals who were presumed positive with a saliva RT-LAMP test between October 2020 and June 2021 (a total of 9 participants). We have revised the following text to clarify sample types.

   Revised Text:

   Line 673: "Individuals who tested positive by **saliva** RT-LAMP were directed to undergo a follow-up confirmatory **nasal** RT-qPCR within 24-48 hours."

3. Also, why were only 8 of the 136 followed up with confirmatory RT-qPCR testing. Could there be a risk of FP results as the NPA was not adequately evaluated in the validation.

   We would like to clarify that 136 refers to the number of positive tests seen during the time period described in the manuscript from a different testing provider on campus, all of which

were RT-qPCR. The number of presumed positive tests seen with our RT-LAMP test was 31 (ln 669). All of these individuals were asked to get a confirmatory RT-qPCR test; however, because of differing procedures throughout the pandemic leading to different locations/providers of the confirmatory tests, we only had access to the results of 9 of the confirmatory tests (ln 674). We revised the text to make this more clear:

> "As of December 16, 2021, a total of 24,927 participants at Rice University were tested in the high-throughput RT-LAMP assay. Thirty-eight people had presumptive positive test results, and the remainder of the participants tested presumptively negative. ~~Presumptive positive test results increased dramatically upon the arrival of the delta variant to Houston (S8 Fig), and the trend seen in LAMP testing presumptive positivity was used in part to inform new guidance on masks and gathering size~~. Individuals who tested positive by saliva RT-LAMP, and five percent of individuals who tested negative by saliva RT-LAMP between September 2020 and January 2021, were directed to undergo a follow-up confirmatory nasal RT-qPCR within 24-48 hours. **Data were available for nine** of the 38 confirmatory tests, **which were performed by one of Rice University's contracted COVID-19 testing providers on a self-collected nasal sample.** Eight received a positive confirmatory test result and one received a negative confirmatory test result**. The research team did not have access to laboratory results for the other 22 positive confirmatory tests which were performed by non-Rice affiliated community providers.** All negative validation tests (n=116) were confirmed negative."

Although there is a risk of false positives, we estimate high specificity based on the confirmatory RT-qPCR results of 116 negatives mentioned in our response to point 1. No changes were made to the text.

4. You mention that during the screening period you saw an increase in positives with emergence of Delta variant. What percentage of the screened specimens were found to be presumptive positive during the Delta surge? This may be a reason why you see lower CT values in this cohort compared to hospitalized patients as Delta appears to have a higher viral load.

   The comparison between Ct values for confirmatory nasal RT-qPCR tests in our surveillance population, the surveillance nasal RT-qPCR tests run by a separate testing provider, and the nasal RT-qPCR tests among hospitalized patients (Fig. 7), only includes data through June 2021, which does not include the Delta variant. The caption of Fig. 7 was updated as indicated below to reflect that data goes through June 2021. This is because, due to the organizational changes throughout the pandemic response at Rice University, we only have confirmatory nasal RT-qPCR results available for tests run prior to June 2021, and we do not have Ct values for the samples that coincide with the rise of the Delta variant in Houston. We added a comment to the manuscript regarding test positivity rates during the period of July 1 – November 1, 2021 (roughly the Delta wave in Houston) compared to other time periods.

Revised Text:

“Fig 7. Nasal Ct distributions in surveillance population and hospitalized patient population. (A) Mean, standard deviation, and range of Ct values and (B) frequency distributions of Ct values for confirmatory nasal RT-qPCR tests after an individual tested positive with LAMP **through June 2021** (n=8), for individuals who tested positive as part of Rice University’s surveillance testing program with the testing provider who conducted confirmatory RT-qPCR tests **through June 2021** (n=136), and for all hospitalized patients who tested positive by nasal RT-qPCR (n=35).”

“Presumptive positive test results increased dramatically upon the arrival of the delta variant to Houston (S8 Fig), **which occurred roughly between July 1 and November 1, 2021. The overall positivity rate prior to July 1 was 0.08%; the positivity rate between July 1 and November 1, 2021 was 0.20%. Additional changes over this period of time that might affect positivity rate include changes to campus-wide policies and testing intervals and the number of people on campus; however** the trend seen in LAMP testing presumptive positivity was used in part to inform new guidance on masks and gathering size.”

5. The manuscript is very detailed, but at this point of the pandemic there are several manuscripts detailing results that were found in the optimization of the assay and the most novel aspect of surveliiance testing at Rice is quickly summarized in the results and discussion. My suggestion is to move most of the optimization data to supplemental and create a short summary few paragraphs in either the results or discussion. This would allow you to expand discussion for the validation and surveillance data.

   Thank you for the suggestion, and we agree there are many papers detailing assay optimization methods. From our perspective, understanding the optimization choices that we made within the main text will be helpful for test developers and implementers, and therefore no changes were made to the text.

   Minor Comments

6. Abstract: Add data for > Ct 30

   We updated the abstract text as follows.

   Revised Text:

   Results of the RT-LAMP assay were compared to traditional RT-qPCR for nasopharyngeal swabs, nasal swabs, and saliva collected from a cohort of patients hospitalized due to COVID-19. ~~For samples with Ct <30 by RT-qPCR, positive agreement with RT-LAMP performed using the isothermal fluorimeter was 100% for nasopharyngeal swabs, nasal swabs, and saliva samples.~~ **For all three sample types, positive agreement with RT-**

LAMP performed using the isothermal fluorimeter was 100% for samples with Ct <30 and 69-91% for samples with Ct <40.

7. Introduction: Although it is well written, the introduction could be cut down. With the amount of literature in the field on SARS-CoV-2 I would remove lines 58-66 and could likely combine the first paragraph and the third.

   We shortened the introduction as follows:

   <u>Revised Text:</u>

   The global COVID-19 pandemic caused by the SARS-CoV-2 virus has demonstrated the need for highly accessible diagnostic and surveillance tests. Testing is a critical component of effective pandemic response and is used to identify outbreaks, inform contact tracing efforts, and contain viral spread. Diagnostic testing on individuals with suspected infections is necessary to provide appropriate clinical care and to maintain separation between individuals with and without confirmed SARS-CoV-2 infections, especially in hospital settings. In addition, SARS-CoV-2 transmission by individuals who are asymptomatic requires routine population-level surveillance for effective outbreak containment when rates of community transmission are high. Accordingly, SARS-CoV-2 testing capacity had to scale quickly to meet massive global demands.

   ~~The first diagnostic tests that received Emergency Use Authorization (EUA) from the United States Food and Drug Administration (U.S. FDA) were based on RT-qPCR, the standard-of-care method for detecting viral RNA. Traditionally, RT-qPCR testing requires sample collection into an appropriate medium, RNA extraction, and RT-qPCR reaction assembly prior to running the test on a thermocycler. Shortly after RT-qPCR testing became available across the country, supply shortages of sample collection kits, RNA extraction kits, RT-qPCR reagents, automated laboratory liquid handlers, and thermocyclers were common and unpredictable. Traditional RT-qPCR remains the standard-of-care method for SARS-CoV-2 diagnosis, but given the supply chain challenges and infrastructure required, alternative methods for surveillance were desirable.~~

   Traditional RT-qPCR is the standard-of-care method for SARS-CoV-2 testing and typically requires sample collection into an appropriate medium, RNA extraction, and RT-qPCR reaction assembly prior to running the test on a thermocycler. Given supply chain challenges associated with RT-qPCR reagents and consumables, as well as the infrastructure required for RT-qPCR testing, alternative methods for surveillance were desirable. A few key findings related to SARS-CoV-2 transmission cleared a path for alternatives to traditional RT-qPCR for surveillance testing. First, epidemiologic modeling indicated that test frequency mattered more than test sensitivity

for effective outbreak prevention [1–3]. In addition, data showed “super-spreading”, or transmission from presymptomatic or asymptomatic individuals with high viral loads, was a major driver of outbreaks [4–6]. Finally, test results from alternative specimen types like saliva were shown to correlate well with results from nasopharyngeal swabs and to be better indicators of infectivity [7–11]. Together, these findings informed the most critical goals of a SARS-CoV-2 surveillance program: frequent testing to detect asymptomatic individuals with high viral loads, and the flexibility to use alternative specimen types and methods that avoid the supply chain shortages associated with RT-qPCR.

8. Ln301: is the “was not” a typo, or did you use assent template for minors?

To clarify, when we collected informed consent under our IRB-approved research protocol, we did not enroll minors under the age of 18. When we transitioned to collecting testing authorization forms under our surveillance testing program, we allowed individuals under the age of 18 to sign up for tests. We updated the text to clarify this point.

Revised Text:

To evaluate the performance of the developed RT-LAMP test when implemented in a high-throughput format, members of the Rice University community were offered the opportunity to complete their testing requirement by enrolling in a research protocol that was reviewed and approved by the Rice University Institutional Review Board (2021-28). Eligible participants included Rice University students, staff, or faculty who were not exhibiting symptoms of COVID, had no history of a positive nucleic acid, antigen, or antibody test result for SARS-CoV-2, were 18 years of age or older, and were willing and able to provide informed consent. ~~For minors, consent was not obtained from parents or guardians.~~

Following initial evaluation of the high-throughput RT-LAMP test, the test was piloted and then offered routinely as one of several options available to Rice University individuals for required campus-wide surveillance testing, following guidelines for universities established by the Centers for Medicare and Medicaid Services (CMS) [41]. **For minors, authorization was provided by the minor, parent or guardian.**

Participants visited a dedicated collection site, where staff assisted in completing written informed consent forms during the piloting phase, or written testing authorization forms during the surveillance phase. Participants then provided saliva samples by passive drool into a barcoded, sterile 5 mL tube (MTC Bio or Eppendorf) and placed their barcoded sample into a cooler with ice. Samples remained on ice for up to four hours before they were transported to a high-throughput RT-LAMP lab for testing.

9. As you went back to patients that were positive for the validation, how long was the new collection from time of collection, especially as you continually discuss the viral load vs time course of infection.

We note in the section "*RT-LAMP as a surveillance test*" that confirmatory nasal RT-qPCR tests were run within 24-48 hours of the presumptive positive result in our lab. No changes were made to the text.

Comments from Reviewer #2:

(No Response)
